# Evaluation of the effect of a chicken comb extract-containing supplement on cartilage and bone metabolism in athletes

**DOI:** 10.3892/etm.2012.646

**Published:** 2012-07-24

**Authors:** MASAFUMI YOSHIMURA, YUKIHIRO AOBA, TAIJI WATARI, REI MOMOMURA, KEITA WATANABE, AKIHITO TOMONAGA, MICHITAKA MATSUNAGA, YOSHIMASA SUDA, WOO YOUNG LEE, KATSUHITO ASAI, KAORI YOSHIMURA, TAKASHI NAKAGAWA, TETSURO YAMAMOTO, HIDEYO YAMAGUCHI, ISAO NAGAOKA

**Affiliations:** 1 Graduate School of Health and Sports Science, Juntendo University, Chiba;; 2 Department of Medicine for Motor Organ, Graduate School of Medicine, Juntendo University, Tokyo;; 3 Kitashinyokohama Orthopedic Surgery;; 4 Tana Orthopedic Surgery;; 5 Kanagawa University;; 6 Institute of Physical Education, Keio University, Kanagawa;; 7 Everlife Co., Ltd., Fukuoka;; 8 Total Technological Consultant Co., Ltd.;; 9 Department of Host Defense and Biochemical Research, Graduate School of Medicine, Juntendo University, Tokyo, Japan

**Keywords:** hyaluronan, cartilage and bone metabolism, biomarker, supplementary diet, athlete

## Abstract

In a previous study, we revealed that a commercially available product of dietary supplement containing a chicken comb extract (CCE), which is rich in hyaluronan, not only relieves joint pain and other symptoms, but also potentially improves the balance of type II collagen degradation/synthesis in patients with knee osteoarthritis. Since soccer is one of the sports most likely to cause knee osteoarthritis (OA), we evaluated the effect of a CCE-containing supplement on cartilage and bone metabolism in athletes. Fourteen and 15 subjects (all midfielders) were randomly assigned to receive the test product (test group) and the dummy placebo containing only vehicle (placebo group), respectively, for 12 weeks. The daily oral intake of the CCE-containing test product clearly decreased the urinary levels of both C-terminal crosslinked telopeptides of cartilage-specific type II collagen (CTX-II) as a type II collagen degradation marker and the N-terminal telopeptides of bone-specific type I collagen (NTx) as a marker of bone resorption at 12 weeks after the initiation of the intervention. By contrast, no significant reduction was detected in the placebo group at any timepoint during the intervention. These observations indicate that the test product is effective in inhibiting, not only cartilage degradation, but also bone remodeling. Thus, the CCE-containing supplement may be useful for the management of joint health in athletes.

## Introduction

The failure of any component of joints participating in load transmission, particularly bone and articular cartilage, may lead to sport-induced joint injuries. The skeletal challenges appear to vary considerably among different sports. In soccer, the incidence of knee- and ankle-joint injuries is conceivably higher than in any other sports, possibly since soccer athletes expose the lower extremity joints to excessive and repetitive axial loading. Such skeletal injuries are predicted, not only to prevent players from participating in practice and competition, but also to increase the risk of osteoarthritis (OA). Results of various clinical studies have indicated that soccer is one of the sports most likely to cause knee OA (1–4). Gelber *et al* (5) reported that young adults with knee injuries have a considerably increased risk of developing OA.

It is possible that soccer- and certain other sports-related skeletal injuries, as well as acute or chronic physical loadings, are expected to affect the turnover rate of the afflicted parts of the skeleton, particularly the joint tissues, and are detected using systemic biomarker assays. Various molecular markers have been reported as indicators of bone turnover and cartilage metabolism in patients with bone and joint disorders (6,7). Among the biomarkers that have been extensively used are the C-terminal crosslinked telopeptides of type II collagen (CTX-II) and C-terminal propeptides of type II collagen (CPII) as the cartilage-specific type II collagen (CII) degradation and synthesis markers, respectively. N-terminal telopeptides of bone-specific type I collagen (NTx) have been used as the marker of bone resorption (8). Previously, we revealed that CTX-II, CPII and NTx are significantly or substantially elevated in the urine or sera of young athletes compared with non-athlete controls (9).

A commercially available product of dietary supplement containing a chicken comb extract (CCE), which is rich in hyaluronan, (Kojun^®^) has been shown relieve joint pain and other symptoms, as well as to potentially improve the balance of CII degradation/synthesis in patients with knee OA (10,11). Based on these observations, we hypothesized that the CCE-containing supplement product (test product) has an effect on the three biomarkers in athletes. Therefore, in this study, we evaluated the effect of a CCE-containing supplement on cartilage and bone metabolism in athletes.

## Materials and methods

### Subjects

The study was approved by the Human Experimentation Ethics Committee of Juntendo University (Japan) and informed consent was obtained from the participants. A total of 66 collegiate athletes belonging to three different intercollegiate soccer teams were recruited to participate in the study. It was expected that the rate of skeletal injuries and the levels of soccer-related activities or physical loadings are influenced by the position of players within the team and/or on the pitch. These levels were predicted to be higher in the midfielder position compared to any other player positions, including the forward, defender and goal keeper, since midfielders cover a longer distance than the players of other positions (12). For this reason, only midfielders were selectively analyzed in this study.

### Intervention and subject group assignment

The test product was a 300-mg capsule preparation consisting of 157.5 mg of CCE, of which approximately 4.5 mg was hyaluronan, 20 mg calcium lactate, 10 mg propolis extract, 4.9 mg chitosan oligosaccharide, 5.0 mg each of vitamins B_1_ and B6, 2.5 mg vitamin E, 2.0 mg ferric pyrophosphate, 0.1 mg vitamin B_12_ and 192.5 mg of vehicle (a mixture of crystalline cellulose, dextrin and fatty acid sugar esters) (11). Fourteen and 15 subjects were randomly assigned to receive 16 capsules/day of the test product (test group) and those of dummy placebo containing only vehicle (placebo group), respectively. The subjects in the two groups were instructed to take allocated capsules at a dose of 8 capsules twice daily for 12 weeks. The study was executed in accordance with the principles of the amended Declaration of Helsinki and the Ethical Guidelines for Epidemiological Research (established by the Japanese Government in 2004) during the 2010 summer-fall soccer competition season.

### Procedures

The urine and blood/serum samples were collected at baseline (before the intervention) and at 4, 8 and 12 weeks after the intervention, and stored at less than −40°C before assay. Urinal CTX-II and NTx were measured using CartiLaps EIA^®^ (Immunodiagnostic Systems, Inc., Tyne & Wear, UK) and Osteomark^®^ (Inverness Medical Japan Co., Ltd., Tokyo, Japan), respectively, and the values were normalized with creatinine (Cr). Serum CPII was measured using Procollagen II C Propeptide ELISA^®^ (Ibex Pharmaceuticals, Inc., Mont-Royal, QC, Canada).

### Statistical analysis

Unpaired and paired Student’s t-tests were used for the between-group comparison and within-group comparison, respectively. Values were presented as the means ± SD. P<0.05 was considered to indicate a statistically significant result.

## Results

Data from 29 subjects (14 and 15 in the test and placebo groups, respectively) who completed the study were evaluated. Demographic characteristics [age, height, weight, body mass index (BMI), blood pressures and pulse rate] as well as biomarker profiles (CTX-II, CPII, CTX-II/CPII ratio and NTx) were not significantly different between the test and placebo groups (Table I).

Table II shows the changes in the biomarker profiles during the intervention. CTX-II levels were clearly reduced from the baseline in the test group with a statistical significance at week 12 (P<0.01), while no significant reduction was observed in the placebo group at any timepoint during the intervention. Unlike CTX-II, CPII levels were elevated significantly from the baseline at all timepoints (weeks 4, 8 and 12; P<0.01 or <0.05). As a result, the ratios of CTX-II to CPII were decreased to a significant level (P<0.01 or <0.05) at weeks 4, 8 and 12 in the test group and at weeks 8 and 12 in the placebo group. Notably, NTx levels were reduced only in the test group, reaching a significant level at week 12 (P<0.01).

**Figure 1:**
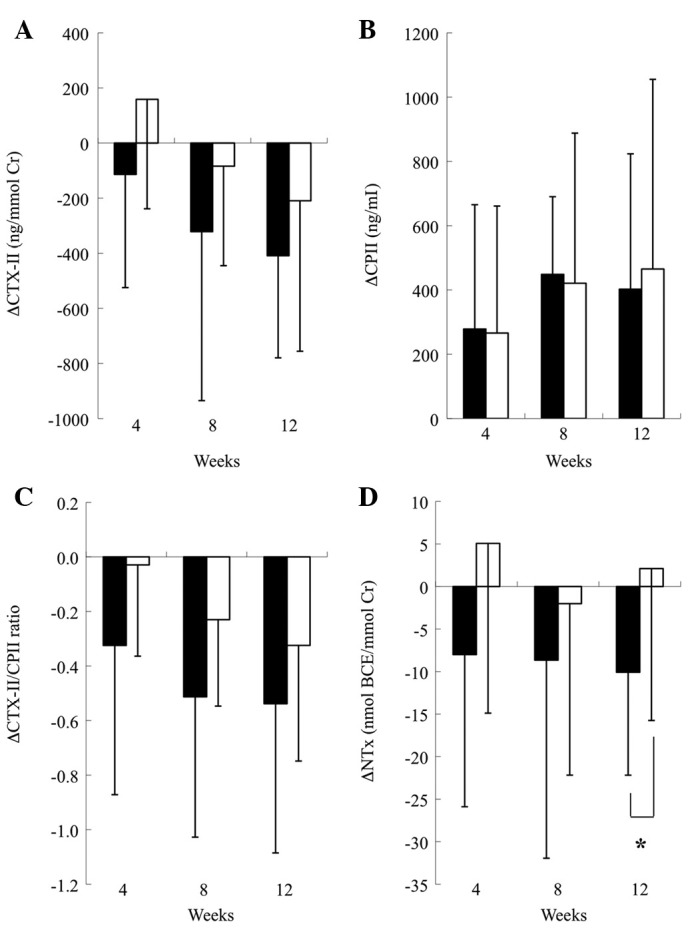
Changes in the levels of (A) urinary CTX-II, (B) serum CPII, (C) CTX-II/CPII ratio and (D) urinary NTx from the baseline in the test group (n=14; black bar) and the placebo group (n=15; whites bar) during the 12-week intervention. Changes in the levels from the baseline are expressed as delta, and are compared between the test and placebo groups. ^*^P<0.05.

Fig. 1 shows the changed values from the baseline in all biomarkers used in this study during the intervention. The changes in the CPII levels were not different between the two groups at any timepoints. By contrast, the changes from the baseline levels of CTX-II, the CTX-II/CPII ratio and NTx were greater in the test group than in the placebo group, and the between-group difference in the NTx was statistically significant at week 12 (P<0.05).

## Discussion

Joint injury and OA are similarly characterized by the remodeling and degradation of cartilage, bone and other joint tissues. Lohmander *et al* (13) reported the release of CTX-II into synovial fluid in human OA and joint injury, which provides the evidence that the integrity of the CII network of cartilage is compromised shortly after joint injury and in arthritis. With regard to OA, accumulating evidence indicates that serum or urinary levels of CTX-II and/or certain other CII degradation markers, including C1, 2C and C2C, increase with the progression of OA (10,11). The presence of NTx in the urine has been evaluated by Bettica *et al* (8) as a risk marker in progressive knee OA and was found to be elevated in patients with the radiographic progression of knee OA. This finding suggests that the progression of OA reflects the changes of both cartilage metabolism and bone turnover, and that the subchondral bone alterations play a role in the pathogenesis of OA. Based on these findings with regard to OA and the similarity of the change in the skeletal marker profiles between OA and joint injury, we have hypothesized that, similar to OA, joint injury is also characterized by cartilage degradation and subchondral bone abnormalities in athletes.

In the present study, we have shown that the daily oral intake of the CCE-containing test product clearly decreases the urinary levels of both CTX-II and NTx at 12 weeks after the initiation of the intervention. The results suggest that the test product is effective in inhibiting cartilage degradation and bone remodeling. In this context, hyaluronan, a putative active component of the CCE-containing supplement, has been reported to stimulate bovine chondrocytes, being effective in maintaining the cell phenotype with increased matrix deposition of glycosaminoglycan and CII (14,15). Although the actual mechanism of this beneficial action on joint injury remains unclear, the CCE-containing supplement may be useful for the management of joint health in athletes.

In conclusion, the oral administration of the CCE-containing supplement decreases the levels of CTX-II and NTx in the urine of young athletes, suggesting a potential use for relieving the joint injury-associated cartilage degradation and bone remodeling.

## Figures and Tables

**Table I:** Baseline data of subjects in the test and placebo groups who completed the study.

Variables	Test group (n=14) (mean ± SD)	Placebo group (n=15) (mean ± SD)	P-value
Age (years)	20.0±1.0	20.0±1.2	1.000
Height (cm)	170.2±4.3	172.6±4.9	0.159
Weight (kg)	63.0±4.4	64.7±5.2	0.359
Body mass index (kg/m^2^)	21.8±1.2	21.7±0.6	0.808
Systolic blood pressure (mmHg)	116.3±11.0	116.1±8.7	0.953
Diastolic blood pressure (mmHg)	63.6±6.4	63.0±7.0	0.821
Pulse rate (beats/min)	60.4±8.7	56.5±8.8	0.251
Urinary CTX-II (ng/mmol Cr)	1,710±883	1,265±1,096	0.242
Serum CPII (ng/ml)	1,544±458	1,498±438	0.784
Urinary CTX-II/serum CPII ratio	1.25±0.81	0.88±0.69	0.195
Urinary NTx (nmol BCE/mmol Cr)	66.8±19.4	60.0±30.1	0.479

BCE, bone collagen equivalent; CTX-II, C-terminal crosslinked telopeptides of cartilage-specific type II collagen; CPII, cartilage-specific type II collagen; NTx, N-terminal telopeptides of bone-specific type I collagen; Cr, creatinine.

**Table II:** Changes in the levels of urinary CTX-II, serum CPII, the CTX-II/CPII ratio and urinary NTx during the 12-week intervention period in the test (n=14) and placebo groups (n=15).

	Groups	Baseline^a^	Week 4^a^	Week 8^a^	Week 12^a^
CTX-II (ng/mmol Cr)	Test	1,710±883	1,596±852 (−7)	1,388±719 (−19)	1,301±706 (−24)^c^
	Placebo	1,265±1,096	1,424±1,239 (13)	1,182±884 (−7)	1,056±693 (−17)
CPII (ng/ml)	Test	1,544±458	1,823±393 (18)^b^	1,993±396 (29)^c^	1,947±544 (26)^c^
	Placebo	1,498±438	1,764±399 (18)^b^	1,919±384 (28)^c^	1,964±515 (31)^c^
CTX-II/CPII ratio	Test	1.25±0.811	0.93±0.54 (−26)^b^	0.74±0.45 (−41)^c^	0.71±0.38 (−43)^c^
	Placebo	0.88±0.69	0.85±0.75 (−3)	0.65±0.47 (−26)^b^	0.56±0.33 (−36)^b^
NTx (nmol BCE/mmol Cr)	Test	66.8±19.4	58.8±18.5 (−12)	58.1±21.0 (−13)	56.7±17.0 (−15)^c^
	Placebo	60.0±30.1	65.0±32.4 (8)	58.0±18.9 (−3)	62.1±23.0 (4)

a Values are expressed as the mean ± SD and the percentage changes from the baseline are indicated in parentheses. Values are compared between the baseline and the timepoints after the intervention.

b P<0.01;

c P<0.05. BCE, bone collagen equivalent; CTX-II, C-terminal crosslinked telopeptides of cartilage-specific type II collagen; CPII, cartilage-specific type II collagen; NTx, N-terminal telopeptides of bone-specific type I collagen; Cr, creatinine.
